# Nearby and non-nested genes in the human genome have more similar genotype tissue expression

**DOI:** 10.1371/journal.pone.0307360

**Published:** 2024-09-18

**Authors:** Jiahong Dong, Stephen Brown, Kevin Truong

**Affiliations:** 1 Edward S. Rogers, Sr. Department of Electrical and Computer Engineering, University of Toronto, Toronto, Ontario, Canada; 2 Institute of Biomedical Engineering, University of Toronto, Toronto, Ontario, Canada; Concordia University Irvine, UNITED STATES OF AMERICA

## Abstract

Neighboring genes within a shared promoter arrangement (i.e. opposite direction with the neighboring ends as the transcriptional start sites) are expected to have a high similarity in genotype tissue expression due to the potential overlap in the promoter region. This raises the question of whether similarity in expression profiles depends on orientation of the neighboring genes and whether there exist thresholds of locality where the similarity diminishes. Thus, in this work, we compared genotype tissue expression profiles at different genomic orientations and localities. Interestingly, there exist gene pairs in the human genome with very high or low expression similarity. Shorter chromosomes tend to have more similarly expressed genes. Also, a cluster of 3 adjacent genes within the average range of 20 to 60 kilobase pairs can have very similar expression profiles regardless of their orientations. However, when genes are nested and in opposite orientations, a lower than expected similarity was observed. Lastly, in cases where genotype tissue expression data does not exist or have low read counts (e.g. non-coding RNA), our identified influencing range can be a first estimate of the genotype tissue expression.

## Introduction

Particular genes are needed for the essential functions for particular tissues (e.g. synapsins for neurons [1] and myosins for muscles [2]), these particular genes often exhibit similar genotype tissue expression profiles. A genotype tissue expression profile refers to the measured amount of RNA transcripts of a gene (typically, measured in transcripts per million (TPM)) expressed by an array of measured tissues (e.g. brain and heart). Many of these expression profiles have been deposited in publicly available databases such as the Genotype-Tissue Expression (GTEx) project that provide a comprehensive collection of gene expression profiles for 54 non-diseased tissues sampled across nearly 1000 individuals [3]. Similar expression profiles can be observed at various genomic localities. Genes on different chromosomes can exhibit similar expression profiles, for example: CDK1 (found in chromosome 10), CCNA2 (chromosome 4), and CCNB1 (chromosome 5) [4]; COL1A1 (chromosome 17) and COL1A2 (chromosome 7) [5]; EGFR(chromosome 7) and ERBB2 (chromosome 17) [6]. Genes within the same chromosome can also exhibit similar expression profiles, for example: KLK3 and KLK2 (chromosome 19) [7]; IL5 and IL13 (chromosome 5) [8]; OLIG1 and OLIG2 (chromosome 21) [9]. Since these genes are in completely different locations in the genome, the similarity of expression profile is typically explained by the recurrence of similar transcription factor binding sites in their promoters (e.g. REST binding site is often found in the promoters of neuronal genes [10]).

Intuitively, neighboring genes in the shared promoter arrangement (i.e. opposite direction with the neighboring ends as the transcriptional start sites) should have high similarity of expression profile because their promoter regions can potentially overlap, particularly if they are in very close proximity [11–13]. Indeed, genes in the shared promoter arrangement can exhibit similar expression profiles, for example: MTHFR and CLCN6 [14]; DCTN5 and PALB2 [15]; ATM and NPAT [16]. Curiously, some neighboring genes, even those not in a shared promoter arrangement, can also exhibit similar expression profiles as seen in the human kallikrein gene family [17]. This raises the question of whether similarity in expression profiles depends on orientation of the neighboring genes and whether there exist thresholds of locality where the similarity diminishes. To answer these questions, in this study, we used the GTEx data to compare the similarity of expression profiles between all genes in the human genome, between genes within the same chromosome, and finally between neighboring genes at various localities: genes that are nested within each other; genes that overlap in the exons, introns and termination sites; genes that are neighbors at various peripheries (i.e. immediate neighbors and more distal neighbors). Interestingly, we show that the similarity in expression profiles is the highest when genes are close in proximity, but this similarity diminishes quickly as the genes become more distal. Furthermore, relative gene orientation does not typically break this trend, except when genes are nested where nested genes in opposite orientations have reduced similarity.

## Material and methods

### Software and datasets

The human gene expression data used in this paper were obtained from the GTEx Portal (https://gtexportal.org/home/datasets, GTEx Analysis v8 (dbGaP accession phs000424.v8.p2)). The mouse gene expression data were obtained from ASCOT (alternative splicing catalog of the transcriptome) [18]. All data presented in this paper were processed and analyzed using the Python programming language (version 3.9.7).

### Pearson correlation coefficient calculation

To assess the similarity between the expression profiles of two genes, the Pearson correlation coefficient (PCC) is used. This statistical measure quantifies the strength and direction of the linear association between two variables, with a range of -1 to +1. A value of -1 indicates a perfectly negative correlation, while a value of +1 indicates a perfectly positive correlation. When applied to gene expression profiles consisting of 54 tissues, we categorized similarity into four classes following a standard criteria: PCC ≥ 0.6 (very similar), 0.4 ≤ PCC < 0.6 (moderately similar), 0 ≤ PCC < 0.4 (weakly similar), and PCC < 0 (negative) [19]. The expression values in individual tissues (in transcripts per million) for two genes *x* and *y* can be denoted as *x*_*i*_ and *y*_*i*_, respectively. Let the mean values of *x* and *y* be denoted as *u*_*x*_ and *u*_*y*_, respectively. The PCC is then calculated using the following formula.


$$PCC=\frac{\sum_{i}^{54}\left(x_{i}-u_{x})(y_{i}-u_{y}\right)}{\sqrt{\sum_{i}^{54}{\left(x_{i}-u_{x}\right)}^{2}\sum_{i}^{54}{\left(y_{i}-u_{y}\right)}^{2}}}$$ (1)

### Using permutation test to compare random and real PCC distribution

A permutation test randomly shuffles the data and compares the significance of an effect to the distribution of effects observed under a null hypothesis. Permutation tests are non-parametric which do not assume a specific data distribution. It is used here to determine whether the 95th percentile of real PCC values is significantly greater than that among randomly generated PCC values. The null hypothesis is that the 95^th^ percentile of real PCCs is no greater than that of the randomized PCCs. The alternative hypothesis is that the 95^th^ percentile of real PCCs is greater than that of the randomized PCCs. The p-value is the proportion of times the 95^th^ percentile of the randomized PCCs is greater than or equal to the real 95^th^ percentile. This represents the probability of seeing a result as extreme as or more extreme than the observed one, assuming the null hypothesis is true. To estimate the p-value, we do the following:

- Determine the 95^th^ percentile of the real PCC values, and this is the test statistic.
- Set a counter to keep track of how many times the randomized 95^th^ percentile is as or more extreme than the real 95^th^ percentile.
- For each of a total 1,000 iterations, generate 10,000 random expression profiles (i.e. roughly the number of genes in the human genome), calculate the randomized 95^th^ percentile. If the randomized 95^th^ percentile is greater than or equal to the real 95^th^ percentile, increment the counter.
- The p-value is calculated by dividing the final counter value by the total number of permutations plus one.

If p-value is small (e.g. below 0.05), reject the null hypothesis and conclude that the 95^**th**^ percentile of the real PCCs is significantly greater than that of the random PCCs, suggesting that the expression profiles have stronger correlations than expected by chance. If p-value is large, the null hypothesis is not rejected, indicating that the 95^**th**^ percentile of the real PCCs is not significantly different from what would be expected by random chance. A similar approach is repeated for the 5^**th**^ percentile.

## Results and discussion

### Determining the similarity of expression profile between all genes

**Fig 1:**
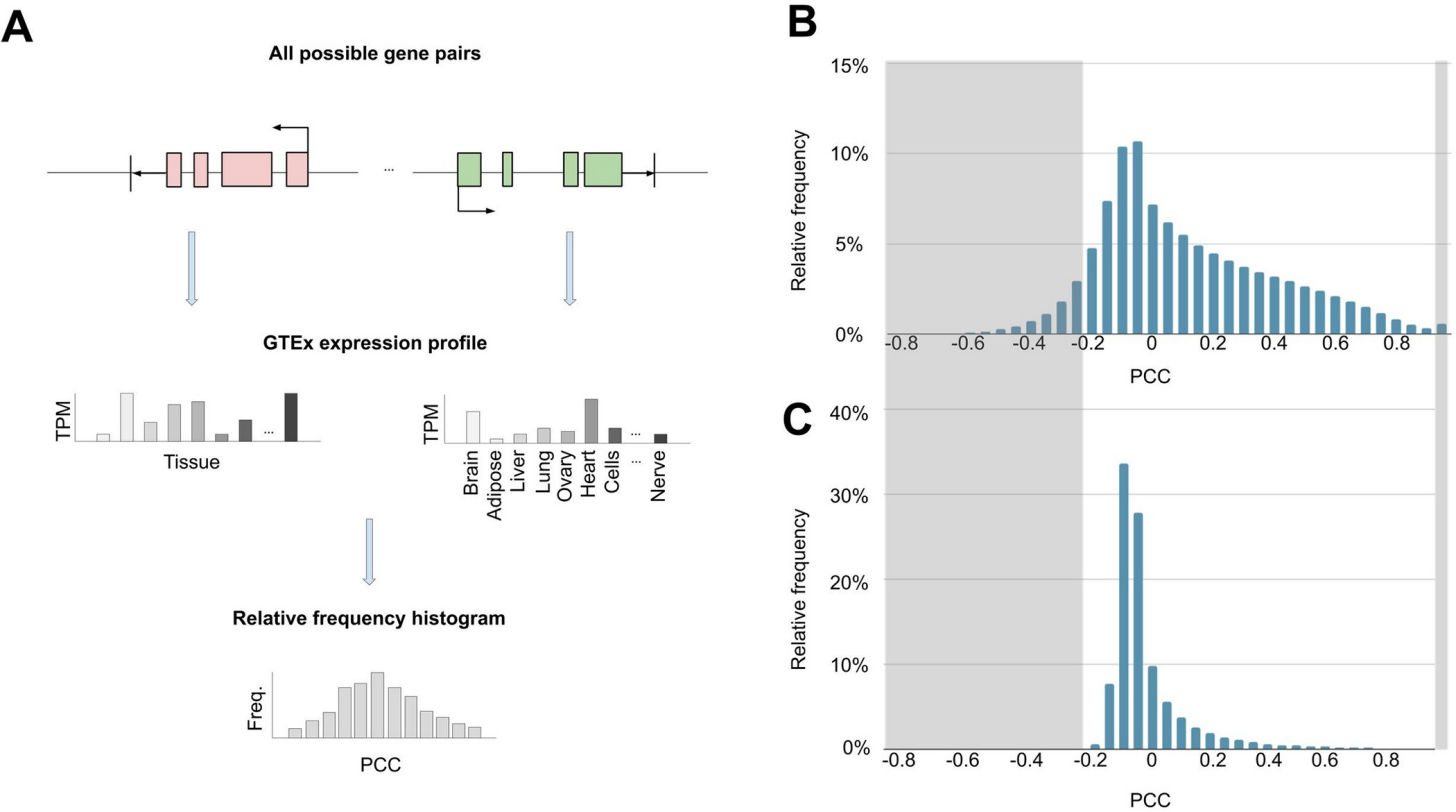
The PCC distribution is calculated between all pairs of genes. **(A)** The gene structure and GTEx expression profiles of two example genes. The red gene denotes the first gene on chromosome 1 (the first chromosome), while the green gene represents the last gene on chromosome Y (the last chromosome). By employing a one-against-all strategy, a PCC is computed by comparing the expression profiles of the red gene and every gene up to and including the green gene. The PCCs are then tabulated into a relative frequency histogram. **(B**) A relative frequency histogram of the real PCC values. **(C)** A relative frequency histogram of the randomly generated PCC values.

When the genotype tissue expression (hereafter, profile) was compared between all possible gene pairs, there was an unexpectedly large number of gene pairs with very different (i.e. Pearson correlation coefficient below -0.2, the low PCC set) and with very similar profiles (i.e. Pearson correlation coefficient from 0.95 to 1, the high PCC set) (Fig 1B). To quantify the similarity between two profiles, the Pearson correlation coefficient (PCC) was used. To show the effectiveness of PCC in measuring similarity, we applied it to gene pairs with known similar expression profiles. The results showed high PCC values: CDK1, CCNA2, and CCNB1 (0.98); COL1A1 and COL1A2 (0.94); EGFR and ERBB2 (0.71); KLK3 and KLK2 (0.99); IL5 and IL13 (0.99); OLIG1 and OLIG2 (0.99); MTHFR and CLCN6 (0.64); DCTN5 and PALB2 (0.72); ATM and NPAT (0.85). To establish the probability distribution of PCC (hereafter, PCC distribution) between gene pairs in the human genome, we calculated the PCCs between the profiles of every possible gene pair in the human genome (Fig 1A). As a result, we obtained a total of 157,522,375 PCC values, showing an overall right-skewed distribution with most values being positive and a spike in the last bin (i.e., 0.95 < PCC ≤ 1, count = 854,563) (Fig 1B and S1 Fig). To determine if the result is an unexpected probability distribution, we repeated the PCC calculation using random profiles. When generating random profiles, a commonly employed method to maintain the original distribution is to randomly select values within the range of original data. However, the presence of extreme values produces “gaps” in the data range where the original data do not occupy. Consequently, values generated from this range may not accurately represent the original probability distribution. To address this issue, we populated each of the 54 tissue expression values by randomly selecting real data from the respective tissue (S3 Fig) and conducted a permutation test to compare the real and random expression profiles. The p-value for both the 5^th^ and 95^th^ percentile was smaller than 0.05, leading us to reject the null hypothesis. This indicates that there are real PCCs significantly greater or smaller than the random PCCs (i.e. the low and high PCC sets), prompting us to explore the potential causes of this phenomenon. Moreover, comparing the random and real PCC distribution, we noticed that the random distribution rarely had values below -0.2, and the spike in the last bin was absent, indicating that the real PCC distribution in the human genome is an unexpected probability distribution (Fig 1C and S2 Fig). This can in part be explained by gene suppression mechanisms and shared transcription factor binding sites.

### Shorter chromosomes tend to have more similarity of expression profile

**Fig 2:**
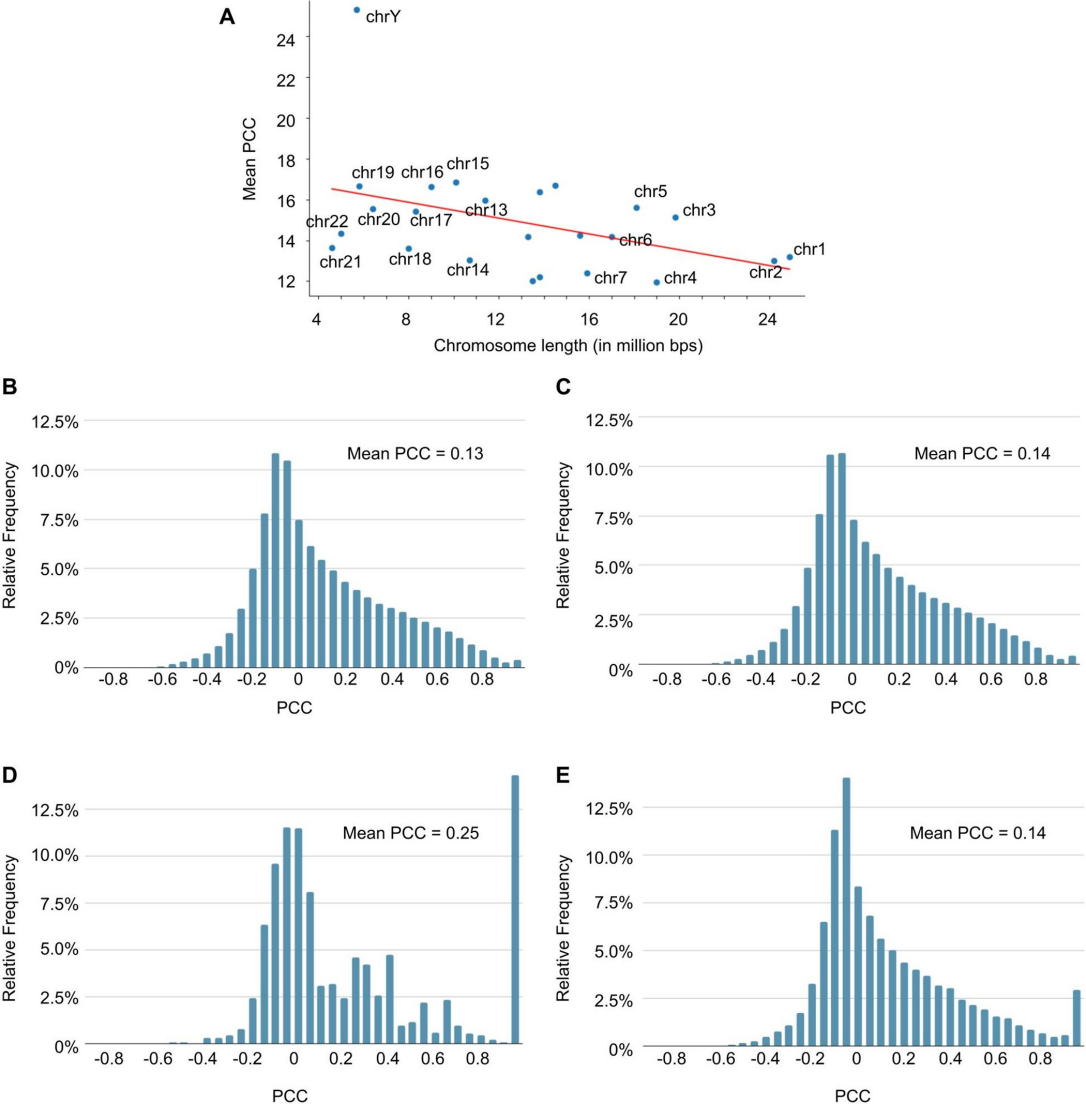
The mean PCCs. **(A)** Scatter plot showing the mean PCC per chromosome versus chromosome length. The line of best fine in red (r^2^ = 0.169). **(B)** A relative frequency histogram showing the PCC values of genes on chromosome 2. **(C)** A relative frequency histogram showing the PCC values of genes on chromosome 2 compared to all other genes in the human genome. **(D)** A relative frequency histogram showing the PCC values of genes on chromosome Y. **(E)** A relative frequency histogram showing the PCC values of genes on chromosome Y compared to all other genes in the human genome.

Chromosomes with more similar profiles tend to be shorter in length (Fig 2). Given that the previously obtained PCC distribution covers genes across the entire human genome, it is of interest to explore how these PCC values are distributed on each chromosome. Linear regression analysis on a scatter plot of chromosomal mean PCC by chromosome length shows that shorter chromosomes are only weakly correlated with higher mean PCC values (r^2^ = 0.169) (Fig 2A). Since the PCC distributions are not normal, we further conducted Kolmogorov-Smirnov (K-S) test to compare the pairwise cumulative distribution functions (CDF) of PCC distributions between chromosomes (i.e. chromosome 1 vs chromosome 2, chromosome 2 vs chromosome 3, etc.) (Fig 2A, S4 and S7 Figs). We then use the p-value as an indicator for understanding the PCC distribution across different chromosomes. First, we ranked chromosomes by their lengths in descending order. Then, for example, suppose the CDF of PCC distribution of the longer chromosome 1 is denoted as *F*(*x*), and the CDF of PCC distribution of the shorter chromosome 2 is denoted as *G*(*x*). The null hypothesis is *F*(*x*) ≤ *G*(*x*) for all *x*, and the alternative hypothesis is therefore *F*(*x*) > *G*(*x*). When *F*(*x*) > *G*(*x*) for all *x*, it implies that the values in *x*_1_ (chromosome 1) tend to be less than those in *x*_2_ (chromosome 2). In other words, chromosome 1 tends to have smaller PCCs than chromosome 2. Since there are 24 chromosomes (chromosome 1–22, X, Y), a total of 276 pairwise comparisons were conducted not including repetitions (i.e. chromosome 1 vs chromosome 2 is the same as chromosome 2 vs chromosome 1). Because many tests were conducted simultaneously, the significance level was adjusted to 0.05/276 for correction. Most comparisons rejected the null hypothesis (242 of 276) suggesting that shorter chromosomes tend to have larger PCC values compared to longer chromosomes. This is reasonable because a shorter chromosome has a greater percentage of genes that are physically closer. However, this tendency does not always hold true. For example, the null hypothesis cannot be rejected when comparing certain chromosome pairs: chromosome 3 and chromosome 4; 8 and 9; 16 and 17; 21 and 22. One possible explanation for this phenomenon is that certain chromosomes may contain more functionally similar genes that are clustered (pathway of genes also found in the same location in the genome than in other chromosomes) [20–22]. While the individual shapes of the PCC distributions may vary across chromosomes, when we compare the PCC distribution chromosome wise, the resulting distribution closely resembles the PCC distribution in Fig 1B (Fig 2C and 2E). Since the PCCs continue to show variations among genes within the same chromosome, we wondered about the possibility of finding a proximity clustering threshold. This threshold would imply that genes within a specific range have more similar profiles, and therefore, higher PCCs.

### Genes in a neighborhood have more similarity of expression profile

**Fig 3:**
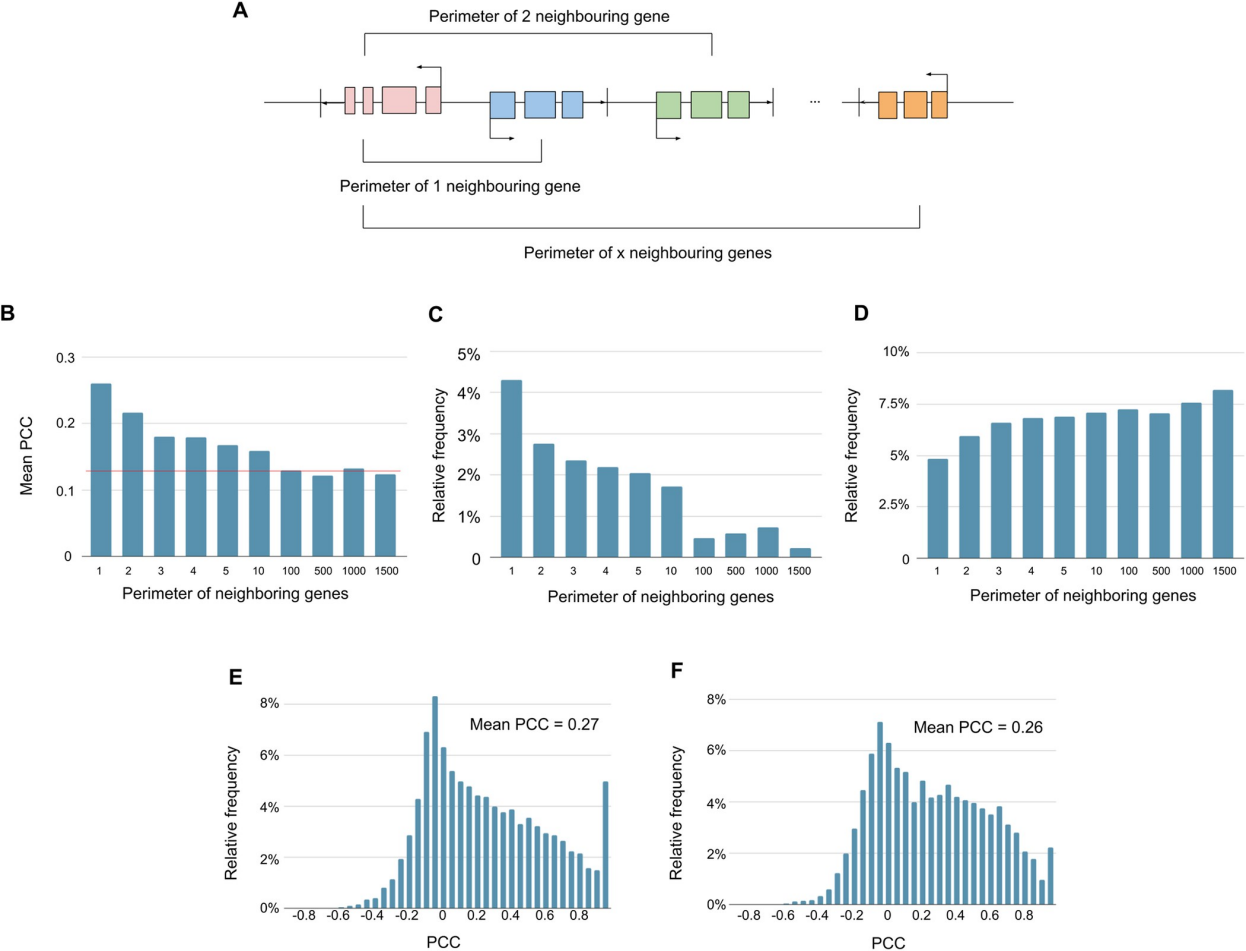
Defining the perimeter of neighboring genes. **(A)** Given the red gene, the blue gene is included in its perimeter of 1 neighboring gene. The orange gene is included in its perimeter of *x* neighboring genes. **(B)** Mean PCC decreases when increasing the perimeter of neighboring genes. The red line is the mean PCC value of all possible gene pairs in the genome. **(C)** The number of PCC > 0.95 (high PCC set) when increasing the perimeter of neighboring genes. **(D)** The number of PCC < -0.2 (low PCC set) when increasing the perimeter of neighboring genes. **(E)** A relative frequency histogram of PCC values of non-shared promoter gene pairs. **(F)** A relative frequency histogram of PCC values of only shared promoter gene pairs.

Genes located in close proximity of 1 to 3 neighboring genes often show more similar expression profiles (Fig 3). We defined gene “proximity” to be the perimeter of *x* neighboring genes relative to that gene on a chromosome (Fig 3A). As *x* can be any positive integer, we investigate similarity at various proximity intervals (i.e., 1,2,3,4,5,10,100,500,1000,1500). For each perimeter interval of *x*, we observed the mean PCC decreased as the perimeter of neighboring genes increased, indicating there is a heightened similarity between expression profiles from close proximity (Fig 3B). This phenomenon can also be verified by repeating the K-S test, indicating that genes in closer proximity tend to have larger PCC values compared to further apart. When *x* = 1, 2, and 3, the mean PCC are noticeably higher than those for other perimeters, indicating that proximity similarity is strongest within a perimeter of 3 neighboring genes (Fig 3B). Moreover, this finding is further supported by a one-way ANOVA test followed by a post-hoc Tukey’s HSD test, as the sample sizes for each proximity interval are sufficiently large. The null hypothesis for the HSD test is that the distributions underlying each proximity interval have the same mean. Beyond *x* = 3, the null hypothesis cannot be rejected, suggesting that the mean PCCs are not significantly different for proximities greater than 3 neighboring genes. For the high PCC set, the number of gene pairs with high PCC values decreases as the perimeter increases (Fig 3C). Similarly, for the low PCC set, the number of gene pairs with low PCC values increases as the perimeter increases (Fig 3D). This is expected since the effect of a transcription factor (i.e. activation or repression) should be consistent (i.e. the transcription factor should not simultaneously be both activator and repressor). In the case of *x* = 1, there is a potential for a shared promoter as defined by two neighboring genes with diverging transcriptional start sites (TSSs) (Fig 3E and 3F). Since genes with shared promoters are known to be often co-expressed, it might be the reason for the higher mean PCC at *x* = 1. To verify, we excluded all gene pairs with potential shared promoters and repeated the tests. Surprisingly, we reached the same conclusions: the strongest proximity similarity is within a perimeter of 3 neighboring genes, and beyond this perimeter, the mean PCCs are not significantly different. This indicates that shared promoter influence on gene expression similarity is absent. Thus, genes in close proximity can have similar expression profiles even without a potential shared promoter. Another genome where tissue-genotype expression data is publicly available is the mouse genome. Our findings suggest that this proximity-based phenomenon extends beyond the human genome, as we also observe a similar trend, with the strongest proximity similarity observed under a perimeter of 3 neighboring protein-coding genes (S5 Fig). The apparent high correlation of promoter activity within close proximity even when genes share no common promoter region suggests that promoter activity is not solely dependent on transcription factor binding to the sequences upstream of the TSS. Our results could potentially be consistently explained by chromatin regulation. For instance, if genes in close proximity (i.e. 1 to 3 genes) have relatively constitutive expression but closed chromatin, there will be no gene expression. If the chromatin is regulated to be open, then all genes in the proximity will have gene expression. Thus, to fully understand promoter activity, it might be critical to also understand how open chromatin is differentially regulated between tissues.

### Nested genes have similarity of expression profile that is orientation dependent

**Fig 4:**
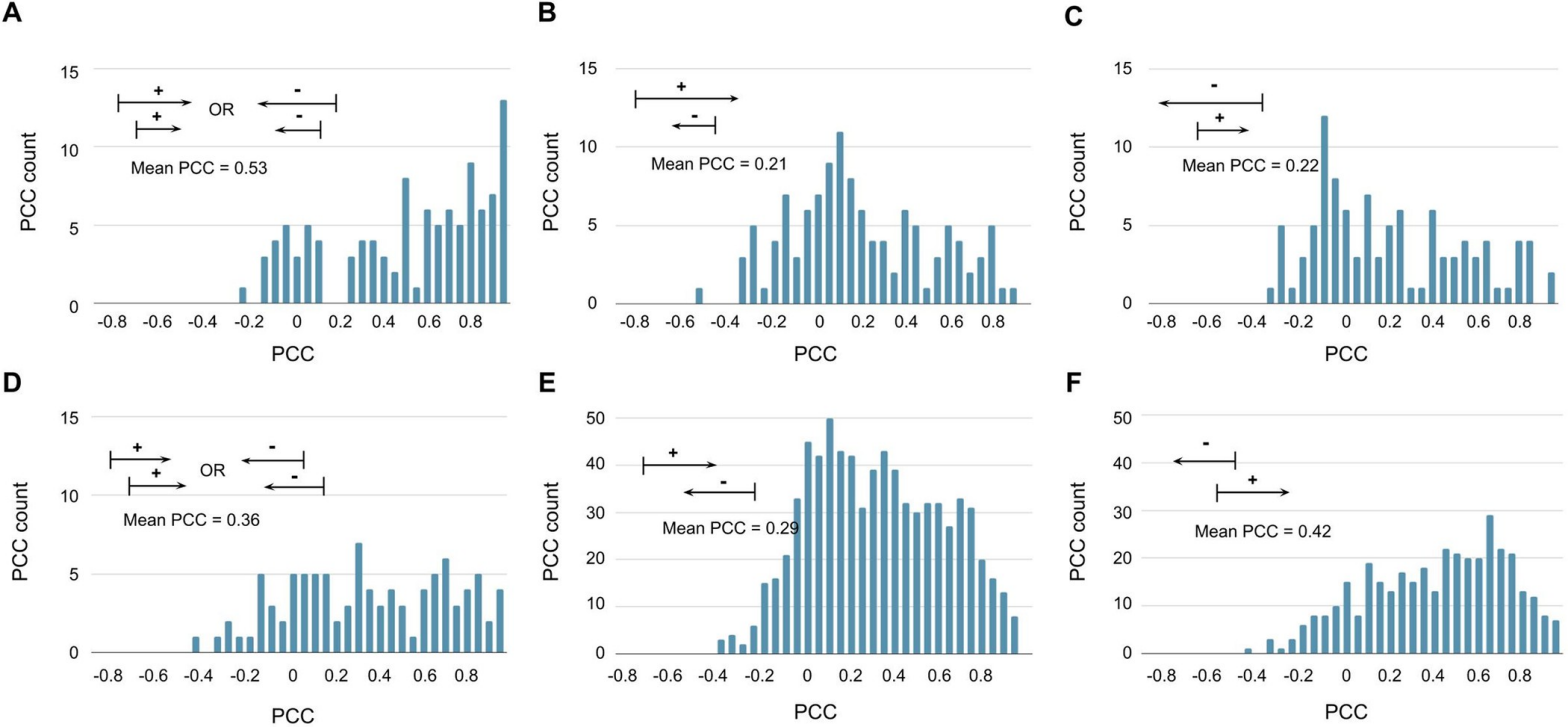
Gene orientations affect similarity in expression profiles. Diagrams showing the orientations are presented in each subfigure. The bar represents the TSSs and the arrow represents the termination sites. **(A)** Nested genes in the “++ or—” orientation. **(B)** Nested genes in “+-” orientation. **(C)** Nested genes in “-+” orientation. **(D)** Overlapping genes in the same orientation “++ or—”. **(E)** Overlapping genes in “+-” orientation. **(F)** Overlapping genes in “-+” orientation.

In gene pairs with perimeter of 0 (i.e. overlap or nested), the mean PCC is highest (Fig 3B) but interestingly, nested genes with the opposite orientation have lower PCC values (Fig 4). There are 1,538 gene pairs with a perimeter of 0. Among these, 326 gene pairs are nested (i.e. the TSS and termination site of one gene bounds the other), while 1,212 gene pairs are overlapped (i.e. TSS and termination site do not bound each other). As expected, the perimeter of 0 has a mean PCC of 0.34, which is higher than the mean PCC of the perimeter of 1. To account for orientation of the gene pairs, we used “+” and “-” to denote genes on the sense and antisense strand, resulting in a total of four possible cases: “++”, “+-”, “—”, and “-+” (Fig 4). For the nested gene pairs, the “++/—" case (i.e. combining “++” and “—“) (Fig 4A) has a mean PCC of 0.53, compared to 0.21 for the “+-” case (Fig 4B) and 0.22 for the “-+” case (Fig 4C). Using a K-S test to compare the CDFs of PCC distributions, the result suggests that PCC values in the “++/—” case tend to be greater than those in both the “+-” and “-+” cases. Furthermore, a one-way ANOVA test followed by Tukey’s HSD test indicates significant differences between the mean PCC of “++/—" and both “+-” and “-+”, but no significant difference between “+-” and “-+”. Due to the smaller sample size compared to the proximity test, we might not safely assume normal distribution based on the central limit theorem. Therefore, we also conducted a non-parametric Kruskal-Wallis H-test followed by Dunn’s test. The results were consistent, showing significant differences in mean rank sum between “++/—" and both “+-” and “-+”, but not between “+-” and “-+”. This is expected because the cases with opposite orientation will make an mRNA transcript that is complementary and therefore, should have a silencing effect [23–25]. In the overlapping gene pairs, the “++/—" case has a mean PCC of 0.36 (Fig 4D), compared to 0.29 for the “+-” case (Fig 4E) and 0.42 for the “-+” case (Fig 4F). The K-S test suggests that PCC values in the “++/—” case tend to be less than those in the “-+” case, but greater than those in the “+-”case. Additionally, the “-+” case tends to have greater PCC values than the “+-” case. Both the ANOVA and Kruskal-Wallis tests indicate that the three overlapping cases are all significantly different from each other, suggesting distinct distributions for each case. Additionally, it seems like the overlapping “-+” case violates our hypothesis where complementary sequences have silencing effects but note that the TSSs are closer in the “-+” case. This could partially behave as a shared promoter, which could increase the similarity in PCCs, suggesting a competing effect. Since the mean number of base pairs that overlap in the transcripts of the case of “+-” and “-+” is 4,914 and 3,191, respectively, the “+-” is more complementary and therefore, should have more silencing effect. Overall, our results suggest that orientations indeed affect the similarity of the expression profile, where same orientations are more likely similar and opposite more likely different.

### Genes within 20k to 60k bps have more similarity of expression profile

**Fig 5:**
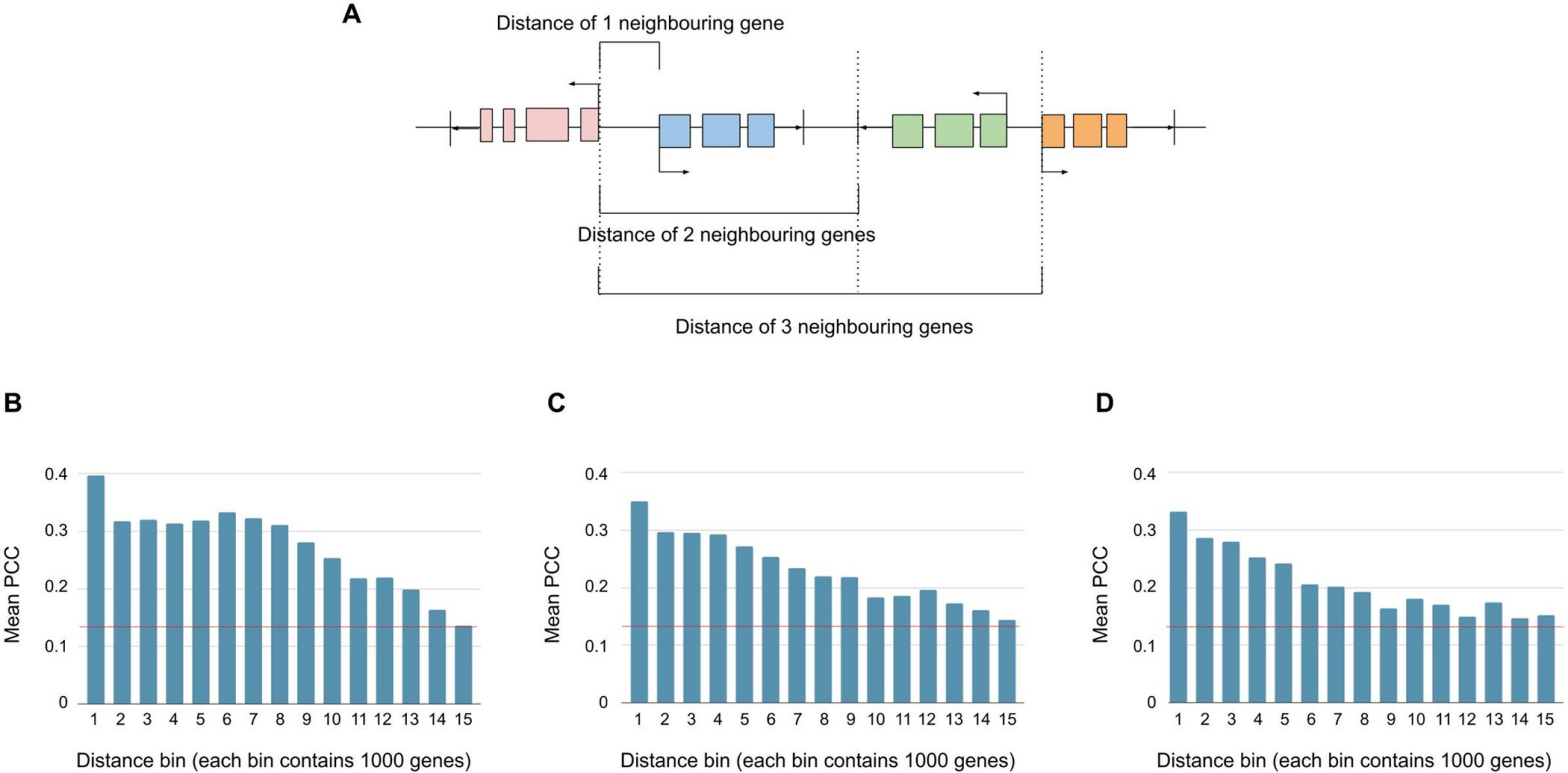
Defining the distance of neighboring genes. **(A)** Given the red gene, the distance between its TSS and the TSS of the blue gene is called the distance of 1 neighboring gene. The distance between the TSSs of the red and green gene is called the distance of 2 neighboring genes. **(B)** Distance versus mean PCC of 1 neighboring gene. The red horizontal line indicates the mean PCC for all gene pairs. **(C)** Distance versus mean PCC of 2 neighboring genes. **(D)** Distance versus mean PCC of 3 neighboring genes.

Converting proximity by perimeter to bps, genes located within 20k to 60k bps often show more similar expression profiles (Fig 5). For each gene pair within a perimeter, the distance between them was calculated relative to their orientation; if genes were in the same orientation, the length was the difference between the termination site and the TSS; if genes were in the opposite orientation, the length was the difference between the TSSs or the termination sites (Fig 5A). The genes are grouped into bins of 1,000 pairs each. The first bin contains the 1,000 gene pairs with the shortest distances between them. Similarly, the second bin contains the next 1,000 gene pairs with the shortest distances, and so on. The mean PCC of each bin is then calculated. This process was repeated for perimeter 1, 2 and 3 to determine the range for proximity threshold (in bp). Generally, the mean PCC decreases with increasing distance between gene pairs. Since the expected mean PCC is 14, most of the mean PCC range is covered within the 9^th^ bin for *x* = 1; for *x* = 2, at the 6^th^ bin; and for *x* = 3, at the 4^th^ bin. The corresponding average distances for these bins are 25,058 bps, 47,096 bps, and 61,789 bps, respectively. Thus, the greatest influence of expression similarity is approximately within 20k to 60k bps.

### RNA genes with existing read counts have similar expression profile to neighboring protein coding genes

**Fig 6:**
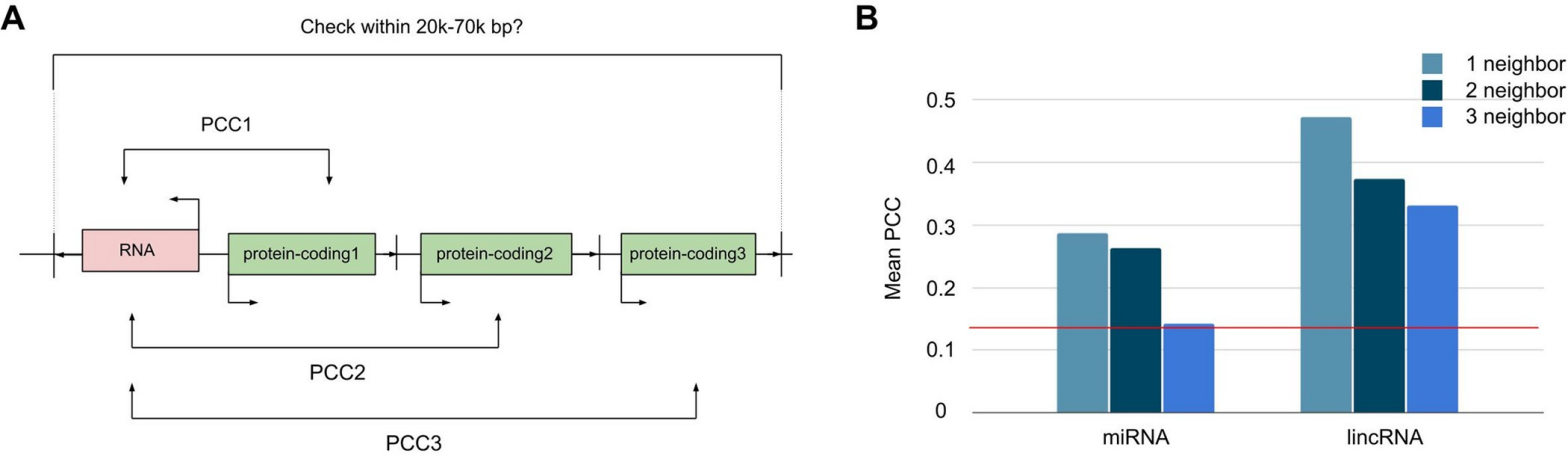
RNA genes have similar expression profiles with up to 3 neighboring protein-coding genes. **(A)** Given an RNA gene, a PCC is calculated between each of its three adjacent protein-coding genes, if the RNA read counts exist and they are within the 20k-60kbp range. **(B)** For lincRNA and miRNA genes, a bar graph of the mean PCC between the three adjacent protein-coding genes. The mean PCC of all genes by all genes is indicated by the red line.

While the number of RNA genes that have existing read counts are low, the ones that have sufficient read counts for analysis demonstrate the same trend–neighboring genes have similar expression profiles (Fig 6). There are two categories of RNA genes that are abundantly found in the GTEx dataset: long intergenic non-coding RNA (lincRNA) has 7,268 genes; micro-RNA (miRNA) has 3,197. Unfortunately, read counts only exist for a small subset of these RNA genes: lincRNA has 4,185 genes; miRNA has 119. The median read counts for the RNA genes (i.e. 134 TPM for lincRNA, 13 TPM for miRNA) are much less than the median read count for protein coding genes (i.e. 398 TPM). Nevertheless, the PCC was calculated for each RNA gene between its 3 adjacent neighboring genes, respectively, if they are within the 60k bps threshold (Fig 6A). Regardless of the RNA type (i.e. lincRNA or miRNA), the closer neighboring genes have a higher PCC, and they are all higher than the PCC of all genes by all genes (Fig 6B). Thus, our result can provide a first estimate of the genotype tissue expression of those RNA genes where the genotype tissue expression data does not exist. We also observed a similar proximity effect when comparing RNA genes vs RNA genes. However, due to the limited number of RNA genes with measurable expression profiles and their relatively sparse distribution compared to protein-coding genes, the proximity threshold for expression similarity decreased to two neighboring genes (S6 Fig). Furthermore, we found that different RNA gene types (e.g., miRNA and lincRNA) can have similar expression profiles when located in close proximity. This could potentially be explained by open chromatin affecting the binding of regulatory elements within a certain vicinity.

## Conclusions

When comparing expression profiles across all possible gene pairs, a notable observation emerged, revealing a substantial occurrence of gene pairs exhibiting either very different or very similar profiles. Shorter chromosomes tend to have more similar profiles. Further, genes situated in close proximity at 1 to 3 neighboring genes exhibited heightened similarity in their profiles. Additionally, overlapping or nesting gene pairs exhibited the highest similarity, yet a noteworthy finding indicated that nested gene pairs with opposite orientations displayed a lower than expected similarity. Moreover, when proximity was translated into base pairs, genes located within the range of 20,000 to 60,000 base pairs demonstrated a tendency toward more similar expression profiles. Since there is abundant genotype tissue expression data of protein-coding genes, our study could make inferences about the expression similarity of non-coding genes, particularly defining a range where the influence on similarity is highest. Thinking further, any gene regulation should be similar in that range even if it is not a protein coding gene. Many RNA genes exist in the human genomes with expression profiles that either do not exist or have low RNA read counts [26–28]. This study suggests these RNA genes should have similar expression profiles to protein-coding genes if they are within the influencing range. In short, these findings shed light on gene proximity and orientation as determinants of similarity in expression profile.

## Supporting information

S1 Fig The GTEx expression profiles of two genes exhibiting an extremely positive PCC (>0.95). These data were obtained from the UCSC genome browser.
(TIF)

S2 Fig The GTEx expression profiles of two genes exhibiting an extremely negative PCC (<0.80). These data were obtained from the UCSC genome browser.
(TIF)

S3 Fig The range of expression values in two randomly selected tissues. **(A)** The range of expression values in adrenal gland. Note that the x-axis has been limited to 100 for a clear graph representation. While the maximum value extends well beyond this threshold, the majority of the expression values typically fall within the range of 0 to 1. **(B)** The range of expression values in Adipose—Subcutaneous. The majority of the expression values still fall within the range of 0 to 1. In fact, for the entirety of the 54 tissues that we examined, the majority of their expression values consistently fall between 0 and 1.
(TIF)

S4 Fig The distribution of PCC on each individual chromosome. PCCs were calculated for every possible pair of genes within a given chromosome following a one-against-all approach.
(TIF)

S5 Fig Mean PCC vs. perimeter of neighboring genes in the mouse genome. **(A)** Mean PCC decreases when increasing the perimeter of neighboring genes in the mouse genome, with the strongest proximity similarity observed under a perimeter of 3 neighboring genes. **(B)** A relative frequency histogram of PCC values of only shared promoter gene pairs. **(C)** A relative frequency histogram of PCC values of non-shared promoter gene pairs.
(TIF)

S6 Fig Mean PCC vs. perimeter of neighboring RNA genes. Mean PCC decreases when increasing the perimeter of neighboring RNA genes in the human genome, with the strongest proximity similarity observed under a perimeter of 2 neighboring genes.
(TIF)

S7 Fig Violin plots showing the distribution of PCC values on each chromosome. (TIF)
