# Supplementary figures and images for: Nearby and non-nested genes in the human genome have more similar genotype tissue expression
Source: PLoS One. 2024 Sep 18;19(9):e0307360. doi: 10.1371/journal.pone.0307360 (PMC11410254; doi:10.1371/journal.pone.0307360)

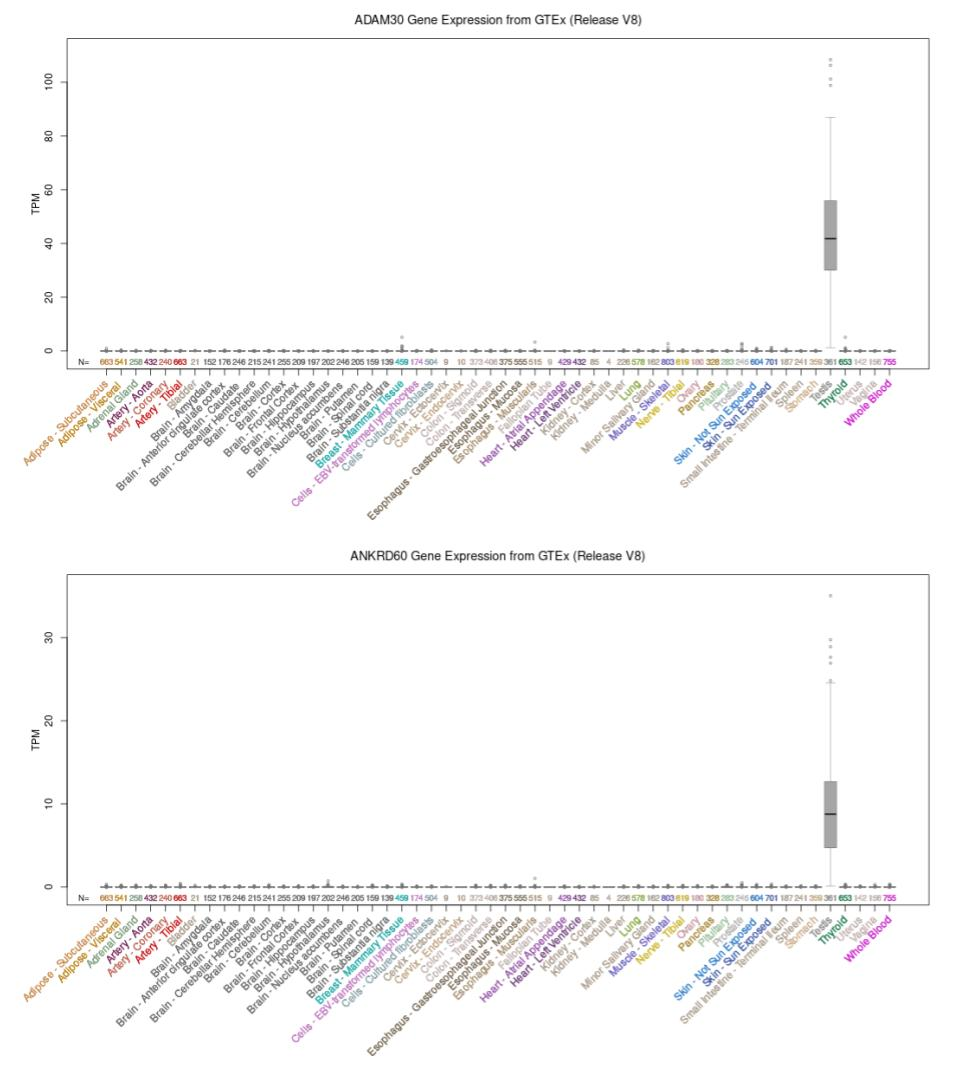

Supplement: S1 Fig — These data were obtained from the UCSC genome browser. (TIF) [file pone.0307360.s001.tif]

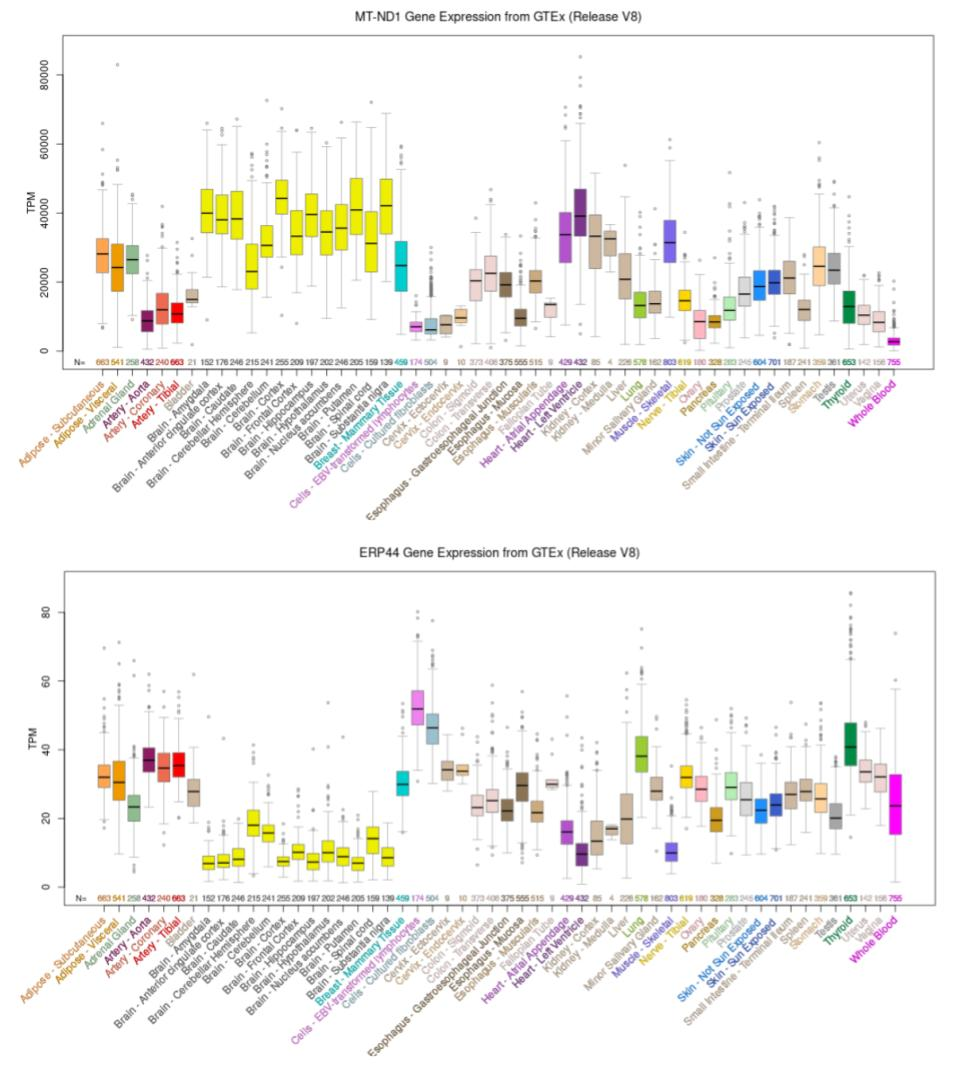

Supplement: S2 Fig — These data were obtained from the UCSC genome browser. (TIF) [file pone.0307360.s002.tif]

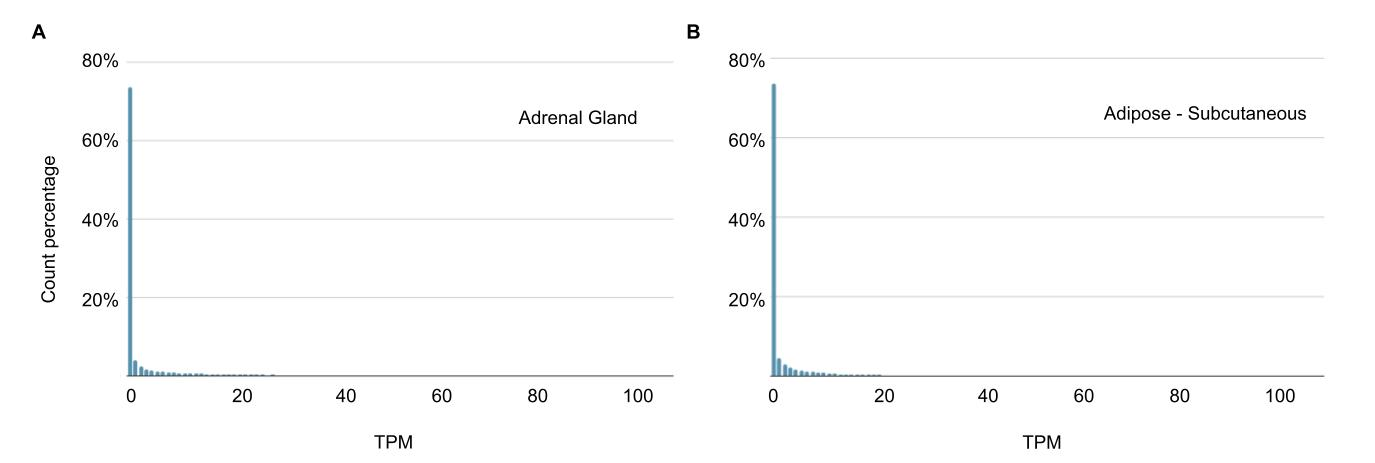

Supplement: S3 Fig — (A) The range of expression values in adrenal gland. Note that the x-axis has been limited to 100 for a clear graph representation. While the maximum value extends well beyond this threshold, the majority of the expression values typically fall within the range of 0 to 1. (B) The range of expression values in Adipose—Subcutaneous. The majority of the expression values still fall within the range of 0 to 1. In fact, for the entirety of the 54 tissues that we examined, the majority of their expression values consistently fall between 0 and 1. (TIF) [file pone.0307360.s003.tif]

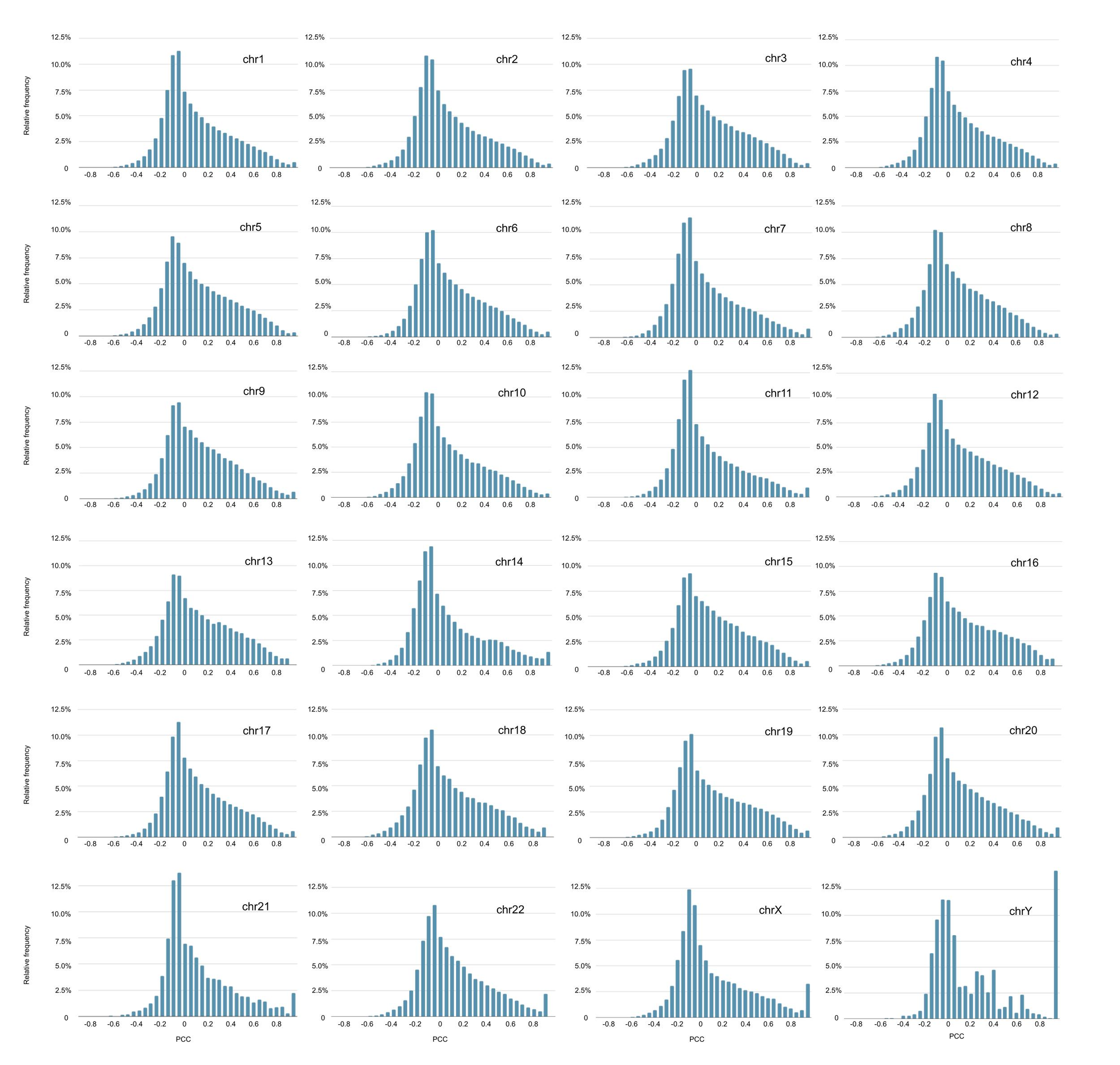

Supplement: S4 Fig — PCCs were calculated for every possible pair of genes within a given chromosome following a one-against-all approach. (TIF) [file pone.0307360.s004.tif]

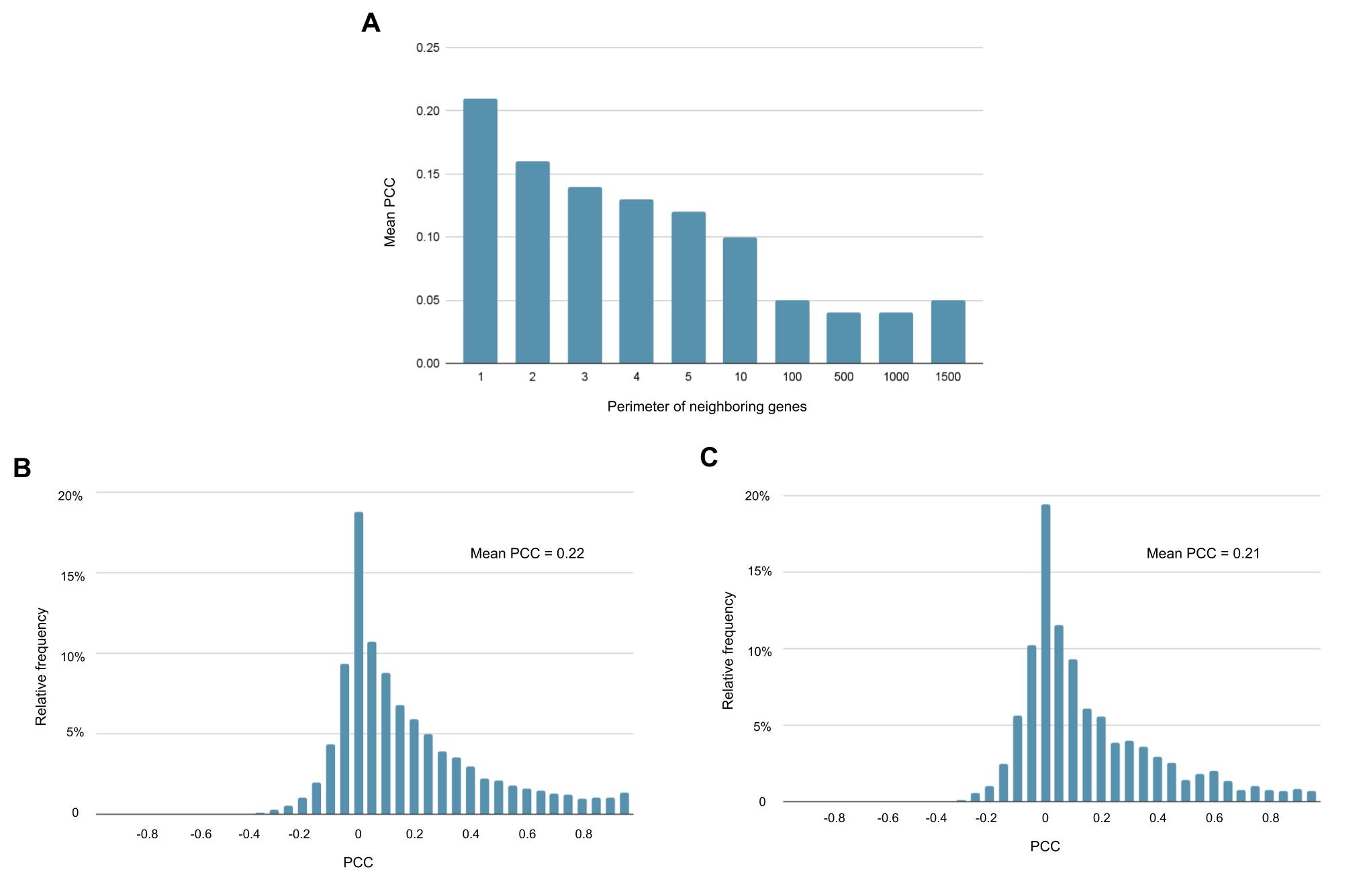

Supplement: S5 Fig — (A) Mean PCC decreases when increasing the perimeter of neighboring genes in the mouse genome, with the strongest proximity similarity observed under a perimeter of 3 neighboring genes. (B) A relative frequency histogram of PCC values of only shared promoter gene pairs. (C) A relative frequency histogram of PCC values of non-shared promoter gene pairs. (TIF) [file pone.0307360.s005.tif]

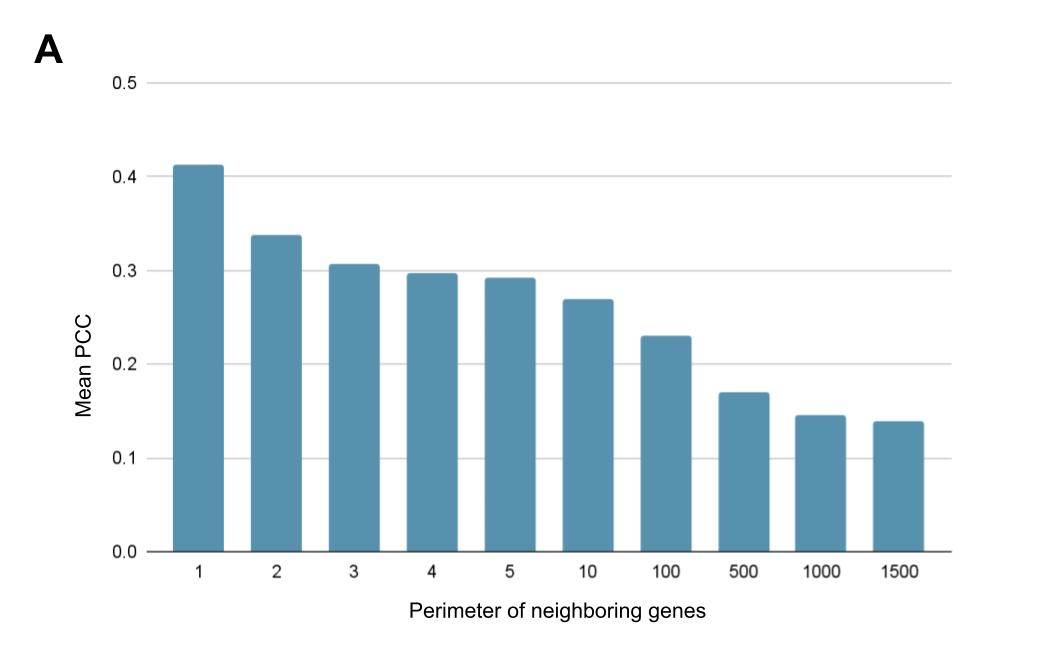

Supplement: S6 Fig — Mean PCC decreases when increasing the perimeter of neighboring RNA genes in the human genome, with the strongest proximity similarity observed under a perimeter of 2 neighboring genes. (TIF) [file pone.0307360.s006.tif]

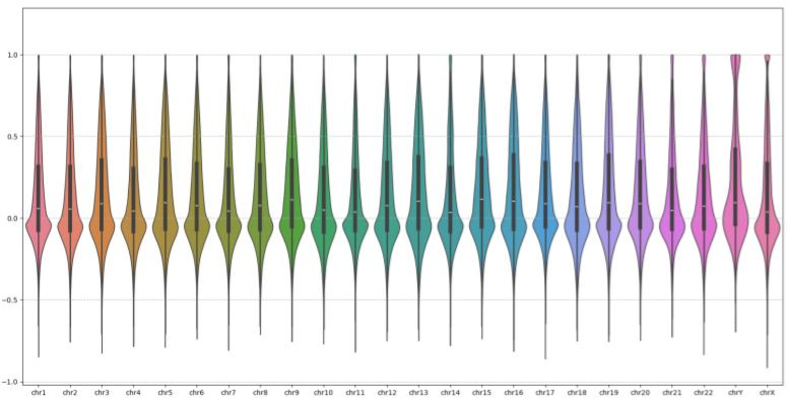

Supplement: S7 Fig — (TIF) [file pone.0307360.s007.tif]
